# Perioperative and palliative systemic treatments for biliary tract cancer

**DOI:** 10.1177/17588359241230756

**Published:** 2024-03-30

**Authors:** Hossein Taghizadeh, Yawen Dong, Thomas Gruenberger, Gerald W. Prager

**Affiliations:** Division of Oncology, Department of Internal Medicine I, University Hospital St. Pölten, St. Pölten, Austria; Karl Landsteiner University of Health Sciences, Krems, Austria; Karl Landsteiner Institute for Oncology and Nephrology, St. Pölten, Austria; Medical University of Vienna, Center for Cancer Research, Vienna, Austria; Medical University of Vienna, Department of Medicine I, Vienna, Austria; Department of Surgery, HPB Center, Health Network Vienna, Clinic Favoriten, Vienna, Austria; Department of Surgery, HPB Center, Health Network Vienna, Clinic Favoriten, Vienna, Austria; Department of Medicine I, Medical University of Vienna, Comprehensive Cancer Center Vienna, Spitalgasse 23, Vienna AT1090, Austria

**Keywords:** biliary tract cancer, immune checkpoint inhibitor, molecular profiling, neoadjuvant therapy, targeted therapy

## Abstract

Due to the fact biliary tract cancer (BTC) is often diagnosed at an advanced stage, thus, not eligible for resection, and due to the aggressive tumor biology, it is considered as one of the cancer types with the worst prognosis. Advances in diagnosis, surgical techniques, and molecular characterization have led to an improvement of the prognosis of BTC patients, recently. Although neoadjuvant therapy is expected to improve surgical outcomes by reducing tumor size, its routine is not well established. The application of neoadjuvant therapy in locally advanced disease may be indicated, the routine use of systemic therapy prior to surgery for cholangiocarcinoma patients with an upfront resectable disease is less well established, but discussed and performed in selected cases. In advanced disease, only combination chemotherapy regimens have been demonstrated to achieve disease control in untreated patients. Molecular profiling of the tumor has demonstrated that many BTC might bear actionable targets, which might be addressed by biological treatments, thus improving the prognosis of the patients. Furthermore, the addition of the immunotherapy to standard chemotherapy might improve the prognosis in a subset of patients. This review seeks to give a comprehensive overview about the role of neoadjuvant as well as palliative systemic treatment approaches and an outlook about novel systemic treatment concept in BTC.

## Introduction

Biliary tract cancer (BTC) represents the second most frequent type of hepatobiliary malignancies and is an aggressive and fatal cancer originating from the epithelium lining of the bile ducts and gall bladder. With a relatively low incidence of below 4/100,000 in the Western world, BTC is deemed as an orphan disease.^1–4^ However, its incidence is clearly increasing. Ouyang *et al.* investigated the burden of BTC in 195 countries between 1990 and 2017 and reported that BTC incidence has increased by 76%, mortality increased by 65%, and disability-adjusted life-years increased by 52% from 1990 to 2017.^5^ Several risk factors have been described to be associated with BTC, including genetic predisposition, smoking, choledochal cysts, cholelithiasis, hepatolithiasis, liver fluke infections in endemic regions, and chronic inflammatory diseases such as primary sclerosing cholangitis, inflammatory bowel disease, non-alcoholic steatohepatitis (NASH), and chronic hepatitis B and C.^6,7^

The entity of BTC includes several subentities, including gallbladder carcinoma, intrahepatic cholangiocarcinoma (iCCA), perihilar cholangiocarcinoma (phCCA), distal cholangiocarcinoma (dCCA), and ampullary carcinoma.^2–4^ Each of these subtypes has a distinct molecular signature highlighting the high spatial heterogeneity in this disease group.^8,9^

Surgical resection of BTC is the only potentially curative therapy option and is only feasible in a localized stage, which is the case in less than 20% of patients with a high rate of recurrence of 60% and a 5-year survival of 8–30%.^10–12^

However, due to the vague symptoms of the disease, it is often diagnosed in an advanced stage not amenable to surgical resection.^13^ For a long time, therapeutic options for metastatic BTC were limited with a dismal 5-year survival rate of only 2%.^14^

Recently, molecular profiling and the development of new drugs with innovative mode of actions, such as immune checkpoint inhibitors and small molecular agents as targeted therapies have enriched the therapeutic armamentarium and – at the same time – increased the complexity of BTC management.^15^

In this review, we give a detailed overview about curative treatment strategies of BTC and discuss the palliative therapies, including the immunotherapy and the molecular-guided targeted drugs.

## Neoadjuvant therapy in cholangiocarcinoma

### Rationale for neoadjuvant therapy

The rationale for the use of neoadjuvant treatment in cholangiocarcinoma is based on several concepts: First, it is postulated that systemic therapy prior to surgery may achieve an eradication of potential micrometastatic disease and consequently a decrease of early recurrence. Additionally, it may help identify patients, who are more prone for developing a progressive disease, which renders them unsuitable for a surgical approach. Another argument in favor of neoadjuvant treatment derives from the observation that it may downsize the primary tumor and thus improve margin-negative resection rate. Furthermore, as patients sometimes experience a delay of adjuvant chemotherapy initiation due to postoperative complications or poor performance status after major liver surgery, preoperative treatment may be considered as an alternative way to increase the receipt of systemic therapy. However, despite these theoretical advantages, it still remains unclear whether neoadjuvant therapy in resectable cholangiocarcinoma is actually associated with a more favorable outcome compared to those, who receive upfront surgical resection. To date, there are no conclusive results from large prospective studies available as of yet, although retrospective analyses have shown a trend toward a survival benefit in patients undergoing neoadjuvant therapy followed by surgery. With the advent of novel treatment strategies for cholangiocarcinoma in recent years, particularly in the field of precision medicine and immunotherapy, several prospective clinical trials are in progress with the aim of investigating the use of neoadjuvant chemotherapy combined with other treatment regimens in (borderline) resectable cholangiocarcinoma. In the following paragraphs, we will outline and discuss available data on the use of neoadjuvant therapy in extrahepatic and intrahepatic cholangiocarcinoma, as well as summarize selected prospective clinical trials that are currently ongoing.

### Extrahepatic cholangiocarcinoma

For patients with phCCA and dCCA, radical surgical resection remains the only potentially curative treatment strategy. In phCCA, surgical therapy is based on extended hepatectomy and bile duct resection including lymphadenectomy and hepaticojejunostomy, whereas patients with dCCA usually undergo a Whipple procedure with pancreaticoduodenectomy. Due to the fact that most patients with an extrahepatic cholangiocarcinoma are diagnosed at an advanced stage (i.e. portal vein invasion, infiltration of the biliary ducts, hepatic artery invasion), the majority of available retrospective analyses on neoadjuvant therapy are conducted in this subtype (Table 1). In 1997, McMasters *et al.* performed a prospective study with nine participants (four dCCA and five phCCA, all unresectable), who received preoperative chemoradiation with 5-FU and external beam radiotherapy (EBRT) prior to hepatic resection. Interestingly, three patients had a pathologic complete response (pCR), whereas R0 resection was achieved in all nine eCCA patients. In the phCCA cohort, none of the patients developed a recurrence. However, in all four dCCA patients, recurrent disease was observed shortly after surgery despite R0 resection. This study has been the first one to report an encouraging safety and efficacy of neoadjuvant chemoradiation in patients with eCCA.^16^ In recent years, several small size retrospective studies followed. Among them, a study led by Nelson *et al.* investigated the efficacy of preoperative 5-FU and EBRT ± brachytherapy in 12 unresectable eCCA patients. The authors observed a R0 resection rate of 91.7% with three patients having a pCR and concluded that these results further strengthened the hypothesis that neoadjuvant treatment may enhance the probability of converting unresectable eCCA to a resectable disease.^17^ Likewise, in 2017 Jung *et al.* also conducted a retrospective analysis, in which 12 patients with unresectable phCCA were treated with 5-FU or Gemcitabine and EBRT, reporting a R0 rate of 83.3% and a pCR in two patients.^18^ In addition to that, two Japanese groups focused on the preoperative application of chemoradiation in resectable eCCA. Katayose *et al.* evaluated the combination of Gemcitabine and EBRT in a prospective trial consisting of 24 patients with resectable eCCA, where R0 resection rate was 89.6% (17/19) among operated cases. The authors concluded that neoadjuvant chemoradiation therapy with conventional resections appeared to be effective and well-tolerated treatment approach.^19^ Similarly, Kobayashi *et al.* performed a retrospective analysis of the clinical effects of neoadjuvant combination therapy with full dose Gemcitabine and radiation therapy *versus* surgery alone in 27 upfront resectable CCA cases (1 GBC, 9 phCCA, 17 dCCA).^20^ The results from this retrospective study revealed an improvement of 3-year DFS (78% *versus* 57%) and 3-year overall survival (OS) (85% *versus* 69%) in the neoadjuvant cohort. Nevertheless, large sized randomized clinical trials are warranted to validate these findings and further explore the efficacy of this treatment strategy.

**Table 1.** Selected summary of completed studies evaluating the role of neoadjuvant therapy in cholangiocarcinoma.

Authors	Year	Study type	Number of pts	Resectability	Study arms	Results
McMasters *et al.*	1997	Prospective (non-randomized)	4 dCCA 5 phCCA	Unresectable	5-FU + EBRT	• 100% R0 rate • No recurrence in phCCA • Disease relapse in all dCCA • 3 pCR
Nelson *et al.*	2009	Retrospective	12 eCCA	Unresectable	5-FU + EBRT ± brachytherapy	• 91.7% R0 rate • 3 pCR • mOS 34 months
Kato *et al.*	2013	Retrospective	22 iCCA	Unresectable	Gemcitabine	• 37% conversion to resection • 3 PR, 11 SD, 8 PD • mOS 19.3 months (resected)
Kato *et al.*	2015	Retrospective	39 iCCA	Unresectable	Gemcitabine + Cisplatin	• 26% conversion to resection • 9 PR, 21 SD, 9 PD • mOS 17.9 months (resected)
Katayose *et al.* (NACRAC)	2015	Prospective (non-randomized)	24 eCCA	Resectable	Gemcitabine + EBRT (45Gy)	• 89.6% R0 rate
Rayar *et al.*	2015	retrospective	45 iCCA	Unresectable	Gemcitabine and/or platinum; Y-90 TARE	• 22% conversion to resection • 100% R0 rate • mDFS 19.1 months
Jung *et al.*	2017	Retrospective	12 phCCA	Unresectable	5-FU/Gemcitabine + EBRT	• 83.3% R0 rate • No advantage in DFS and OS: mDFS 26.0 months, mOS 32.9 months
Kobayashi *et al.*	2017	Retrospective	27 (1 GBC, 9 phCCA, 17 dCCA)	Resectable	Gemcitabine + EBRT → surgery *versus* surgery alone	• PR rate 70% • 3-year DFS 78% • 3-year OS 85%
Omichi *et al.*	2017	Retrospective	43 iCCA	Borderline resectable/unresectable	Gemcitabine based therapy	• 100% conversion to resection • 5-year RFS 48%
Sumiyoshi *et al.*	2018	Retrospective	15 (7 iCCA, 8 phCCA)	Unresectable	S-1 + IMRT	• 73.3% conversion to resection • 82% R0 rate • mOS 37 months (resected)
Le Roy *et al.*	2018	Retrospective	74 iCCA	Unresectable	Gemcitabine + Oxaliplatin	• 53% conversion to resection • 18 PR, 33 SD, 23 PD • 31% R0 rate • mOS 24.1 months (resected)
Chaudhari *et al.*	2018	Prospective (non-randomized)	160 GBC	Borderline resectable/unresectable	Gemcitabine based therapy	• 95% R0 rate • ORR 52.5% • mDFS 25 months • mOS 49 months
Sutton *et al.*	2021	Retrospective	10 iCCA	Resectable	Gemcitabine + platinum	• mRFS 13 months • 5-year OS 80% for neoadjuvant cohort (*versus* 37% upfront surgery)

dCCA, distal cholangiocarcinoma; EBRT, external-beam radiotherapy; eCCA, extrahepatic cholangiocarcinoma; 5-FU, 5-fluorouracil; GBC, gallbladder cancer; iCCA, intrahepatic cholangiocarcinoma; IMRT, intensity-modulated radiotherapy; mOS, median overall survival; mDFS, median disease-free survival; mRFS, median recurrence-free survival; ORR, overall response rate; pCR, pathologic complete response; PD, progressive disease; phCCA, perihilar cholangiocarcinoma; PR, partial response; SD, stable disease.

### Intrahepatic cholangiocarcinoma

With regards to iCCA, current evidence is sourced from a limited number of retrospective cases series evaluating the benefits of neoadjuvant treatment mostly in unresectable iCCA patients (Table 1). Similar to other subtypes of CCA, surgery remains the mainstay of curative therapy for iCCA. So far, combined treatment modalities including chemotherapy, radiation, local liver-directed therapies have provided intriguing results in the preoperative setting and form the basis for further in-depth analyses. Two of these studies were conducted by Kato *et al.* with the primary aim of investigating the efficacy of Gemcitabine-based chemotherapy. In the Gemcitabine monotherapy cohort with 22 iCCA patients, 37% of patients were successfully converted to a resectable disease. In the resected group, a mOS of 19.3 months was reported.^21^ In contrast to that, 39 iCCA patients received the combination of Gemcitabine and Cisplatin preoperatively, resulting in a conversion rate to surgery of 26% and mOS of 17.9 months.^22^

Moreover, Omichi *et al.* observed in 43 patients with borderline resectable or unresectable iCCA a 100% conversion rate to resection and a 5-year RFS of 48% following neoadjuvant treatment with Gemcitabine-based chemotherapy.^23^ Interesting results were recently also published by a French group led by Le Roy *et al.*, where 74 initially unresectable iCCA patients received Gemcitabine and Oxaliplatin in the neoadjuvant setting, of which 53% underwent surgery and 31% had a R0 resection. Of note, mOS of patients with unresectable disease treated with a median length of six cycles of systemic therapy followed by surgery was 24.1 months, which is comparable to the mOS of the resectable patient cohort treated with surgery alone (25.7 months). Consequently, similar outcomes were observed in patients with unresectable iCCA undergoing neoadjuvant chemotherapy followed by surgery and iCCA patients with a resectable tumor.^24^

### Ongoing prospective neoadjuvant trials in cholangiocarcinoma

To date, there are multiple clinical trials ongoing investigating the feasibility and efficacy of neoadjuvant systemic therapy in resectable, as well as in locally advanced cholangiocarcinoma (Table 2). An emphasis has been placed on patients with upfront resectable iCCA. The triple combination of chemotherapy with Cisplatin + Gemcitabine + nab-Paclitaxel is currently being evaluated within the phase II study NEOGAP (NCT03579771), where the primary endpoints are the completion of all preoperative and operative therapy and the incidence of adverse events. A preliminary report has been presented at the 2022 annual meeting of the American Society of Clinical Oncology: Of 30 patients in total, 77% completed all preoperative chemotherapy and received curative-intent resection, with 33% developing grade 3 treatment-related adverse events, most commonly neutropenia or diarrhea. As for the response rate, the disease control rate was 90% with 23% partial response, 67% stable disease and 10% progressive disease.^25^ NCT03603834 is another neoadjuvant chemotherapy study, where (potentially) resectable CCA patients receive mFOLFOXIRI preoperatively. The primary outcome of this trial is the rate of overall response evaluated by MR or CT within the time frame of up to 15 weeks.^26^ In contrast, NCT04669496 is a phase II–III clinical trial evaluating the combined use of the PD-1 antibody Toripalimab + GEMOX + Lenvatinib *versus* observation in resectable iCCA patients with high-risk recurrence factor.^27^ After surgical resection, adjuvant chemotherapy consisting of Capecitabine monotherapy will be administered to all patients. The primary outcome is event-free survival within 24 months. Furthermore, a phase II trial initiated in Korea investigates the addition of the PD-L1 checkpoint inhibitor Durvalumab to Cisplatin and Gemcitabine in patients with localized BTC (DEBATE, NCT04308174). The primary outcome measure is R0 resection rate.^28^

**Table 2.** Overview of ongoing prospective neoadjuvant trials in cholangiocarcinoma.

Trial ID/NCT number	Study type	Location	Tumor site	Resectability	Number of pts	Therapy regimens	Primary outcomes	Estimated year of completion
GAIN (NCT03673072)	III	Germany	CCA, GBC	Incidental diagnosis post CHE	300	Cisplatin + Gemcitabine	OS	2024
DEBATE (NCT04308174)	II	Korea	CCA, GBC	Resectable	45	Durvalumab + Cisplatin + Gemcitabine *versus* Cisplatin + Gemcitabine	R0 Rate	2023
NEO-GAP (NCT03579771)	II	USA	iCCA	Resectable	34	Cisplatin + Gemcitabine + nab-Paclitaxel	Completion of all preoperative and operative therapy, incidence of adverse events	2023
NCT03603834	II	Thailand	CCA	Resectable	25	mFOLFOXIRI	Rate of overall response evaluated by MRI or CT (up to 15 weeks)	2023
NCT04506281	II	China	iCCA	Resectable	128	Gemcitabine + Oxaliplatin + Lenvatinib + Toripalimab	Event free survival (18 months)	2023
NCT04669496	II	China	iCCA	Resectable	178	Gemcitabine + Oxaliplatin + Lenvatinib + Toripalimab → surgery → adj. Capecitabine	Event free survival (24 months)	2024
POLCAGB (NCT02867865)	II–III	India	GBC	Advanced (unresectable without evidence of distant metastases)	314	Cisplatin + Gemcitabine *versus* CRT (Gemcitabine) → Cisplatin + Gemcitabine	OS	2027
NCT04523402	II	China	iCCA with high-risk LN metastases	Resectable	100	Gemcitabine + Oxaliplatin	Event free survival (24 months)	2023
NCT04989218	I–II	USA	iCCA with high-risk features	Resectable	20	Gemcitabine + Cisplatin + Durvalumab + Tremelimumab	ORR	2024
NCT05557578	II	China	iCCA with high recurrence risk	Resectable	20	Tislelizumab + GEMOX	ORR, R0 Rate	2024
NCT05672537	II	China	iCCA with high recurrence risk	Resectable	70	Durvalumab + Gemcitabine + Cisplatin	1-year relapse free survival rate	2025
OPT-IN (NCT04559139)	II–III	USA	histologically-confirmed T2/T3 GBC (incidental)	Resectable	183	Gemcitabine + Cisplatin neoadjuvant and adjuvant *versus* Gem/Cis only adjuvant	OS	2023
JCOG1920: NABICAT (jRCTs031200388)	III	Japan	BTC	Resectable	330	Gemcitabine + Cisplatin + S1 *versus* surgery first	OS	NA
NCT04378023	NA	Spain	phCCA	Unresectable	34	EBRT + Capecitabine → Gemcitabine + Cisplatin until transplant	OS at 1, 3 and 5 years	2025

BTC, biliary tract cancer; CCA, cholangiocarcinoma; CRT, chemoradiotherapy; EBRT, external beam radiation therapy; GBC, gallbladder cancer; iCCA, intrahepatic cholangiocarcinoma; mFOLFOXIRI, modified fluorouracil + oxaliplatin + irinotecan; phCCA, perihilar cholangiocarcinoma; ORR, overall response rate; OS, overall survival.

Another interesting trial is the phase II SIROCHO trial (NCT05265208) is the combination of Selective Internal Radiation Therapy (SIRT) with capecitabine in the neoadjuvant setting of resectable iCCA. Similar to NCT04669496, the primary outcome measure is R0 resection rate.

Further, the phase II IMHOTEP (NCT04795661) trial pursues an innovative concept and investigates the efficacy of the immune checkpoint inhibitor pembrolizumab in the perioperative setting of localized deficient mismatch repair/microsatellite instability-high (dMMR/MSI-high) tumors independently of their anatomical origin, including BTC. Primary objective is to evaluate the pathological complete response (pCR) rate defined as ypT0N0 stage.^29^

Until now, there are no phase III trial results available demonstrating effectiveness of neoadjuvant treatment in cholangiocarcinoma. However, just recently the German study GAIN (NCT03673072), a multi-centered, randomized controlled, phase III trial has been initiated. The main target of this study is to examine the superiority of neoadjuvant therapy (preoperative Gemcitabine and Cisplatin, followed by surgical resection and adjuvant chemotherapy) over surgery (plus adjuvant chemotherapy, regimen per investigators choice) in terms of OS. Patients with either incidental gallbladder cancer found after cholecystectomy or resectable extrahepatic and intrahepatic cholangiocarcinoma can be included into this trial.^30^ Furthermore, a phase III trial (JCOG 1920/ NABICAT) of neoadjuvant GCS (Gemcitabine + Cisplatin + S1) *versus* surgery first for resectable BTC has commenced patient recruitment in Japan.^31^ Patients of the experimental arm receive three cycles neoadjuvant GCS prior to surgical resection. OS represents the primary outcome of this trial. Finally, there are currently also few studies underway assessing the effect of conversion chemoradiotherapy in patients with unresectable CCA. For example, POLCAGB (NCT02867865) is a phase II–III clinical trial comparing neoadjuvant Cisplatin + Gemcitabine to 5 weeks of concomitant chemoradiotherapy (Gemcitabine with 45 Gy EBRT) followed by two cycles of Cisplatin + Gemcitabine in patients with an unresectable GBC but without evidence of distant metastases with OS as the primary endpoint.^32^

### Neoadjuvant therapy and liver transplantation in cholangiocarcinoma

The current status of liver transplantation for the treatment of cholangiocarcinoma is based on the concept that liver transplantation might offer advantages over liver resection, with an improved probability of achieving negative oncologic margins, whereas eliminating intrahepatic micro-metastases and resolving any preexisting liver disease at the same time.^33^ The most established protocol to date has been published by the Mayo Clinic Group, employing neoadjuvant chemoradiation followed by liver transplantation as a definitive therapy for patients with perihilar CCA.^34^ Essentially, early stage perihilar CCA patients with <3 cm lesions on imaging without metastasis or lymph node involvement, who are technically unresectable, will undergo endoscopic ultrasound and fine needle aspiration of regional lymph nodes. Those with negative lymph nodes will receive chemoradiotherapy for 5 weeks. External beam radiation therapy, which is accompanied by 5-fluorouracil as a radiation-sensitizing agent, and intraductal radiation therapy will be applied sequentially, followed by oral capecitabine treatment until the patient undergoes liver transplantation. Subsequently, laparoscopic or open abdominal exploration will be performed to exclude metastasis and examine regional lymph nodes prior to liver transplantation. Post-transplantation management includes standard immunosuppressive treatment but no adjuvant systemic therapy. The cumulative dropout rate from the ‘Mayo Protocol’ reaches 46% by 12 months. OS at 5 years is 68% ± 3% and at 10 years is 60% ± 4%. The authors concluded that the majority of deaths following liver transplantation are due to recurrent disease.^34^

In contrast, the current data situation regarding the use of liver transplantation in intrahepatic CCA is not clear and requires further in-depth exploration. Most literature on liver transplantation in iCCA focused on incidentally found tumors on explant livers or in patients who were transplanted for presumed HCC, which does not typically undergo neoadjuvant systemic therapy prior to transplant but rather only locoregional therapy.^35^ Consequently, substantial evidence is lacking to support protocols that combine neoadjuvant treatment with liver transplantation for iCCA. In fact, studies which include neoadjuvant therapy prior to liver transplantation are confined to small case series only. For instance, Lunsford *et al.* assessed 12 patients with non-metastatic unresectable iCCA >2 cm in size, who underwent neoadjuvant gemcitabine and platinum-based chemotherapy for 6 months. Six patients (50%) showed either stable disease or regression and proceeded to liver transplantation. The 5-year OS was reported with 83.3%.^36^ Furthermore, Wong *et al.* performed a similar study with the addition of transarterial chemoembolization and pre-transplant operative staging. From the 18 patients, who started the neoadjuvant therapies, only 5 (27.8%) patients proceeded to liver transplantation. Follow-up at 1 year demonstrated an OS of 80%, whereas recurrence developed in two patients.^37^

All in all, patients with response to neoadjuvant therapy have better outcomes after liver transplantation compared to those without response to neoadjuvant therapy. Although more evidence is needed, there is an increasing opinion in favor of extending the indication of liver transplantation for unresectable iCCA. Based on the consensus statement from the European Network for the Study of Cholangiocarcinoma in 2020, liver transplantation should be considered, particularly in iCCA patients with very early-stage unresectable tumors (⩽2 cm) and concomitant cirrhosis.^38^

In the neoadjuvant setting, it is of utmost importance to preserve liver function in cirrhotic patients, while achieving optimal anti-tumor effect of the systemic therapy. Of note, in iCCA patients with underlying cirrhosis, liver transplantation may be regarded as a potential treatment strategy.^38^

### Adjuvant therapy for cholangiocarcinoma

In terms of adjuvant treatment strategy for BTC, six randomized phase II/III trials investigating the efficacy of adjuvant chemotherapy are currently available. Single-agent capecitabine was established as the standard treatment regimen in the adjuvant setting for BTC based on the results from the BILCAP trial.^39^ Although its primary outcome was not achieved in the intention-to-treat population, it did demonstrate a statistically significant improvement in OS in the per-protocol analysis. Another limitation of this trial was the relatively long enrollment time from March 2006 to December 2014 to include 447 patients.

In contrast, ESPAC-3 was a phase III trial conducted to assess the efficacy of adjuvant chemotherapy using 5-fluorouracil and folinic acid or gemcitabine monotherapy *versus* surgery alone in patients with extrahepatic cholangiocarcinoma and ampullary carcinoma. Significant superiority of adjuvant chemotherapy over surgery alone was not achieved based on the intention-to-treat analysis.^40^ Similarly, gemcitabine-based chemotherapy – either gemcitabine alone in the BCAT study or gemcitabine in combination with oxaliplatin in the PRODIGE-12 – failed to show significant activity in the adjuvant setting.^10,41^ Of note, the randomized phase II STAMP trial, which was currently published by Jeong and Yoo explored the efficacy and feasibility of adjuvant gemcitabine combined with cisplatin (experimental arm) over capecitabine (control arm) in a specific subset of patients with BTC.^42^ Only patients diagnosed with extrahepatic cholangiocarcinoma (both hilar and distal cholangiocarcinoma), who had undergone curative surgery with R0 or R1 resection and who had a positive lymph node status in the surgical specimen were included in the STAMP trial. Despite the fact that the study failed to demonstrate an improvement in outcome in the gemcitabine/cisplatin cohort, the study design was positively highlighted due to its subgroup-specific nature, since the authors decided to focus on one specific subgroup of patients rather than recruiting patients with all types of biliary tract tumors. Finally, it is worth mentioning that the randomized phase III ASCOT study is the only recent positive adjuvant clinical trial.^43^ Single-agent fluoropyrimidine-based therapy with S1 demonstrated significant efficacy and improvement in survival in Asian patients in the adjuvant setting and thus may be considered as a potential new standard of care for resected BTC in the Asian population.

## Palliative therapy in BTC

### SACT in first line without biomarker guidance

Since 2010 until recently, the pivotal ABC-02 phase III trial set the standard first-line treatment for metastatic BTC (mBTC) with a chemotherapy combination therapy of gemcitabine with cisplatin which was superior to gemcitabine monotherapy in terms of median progression-free survival (mPFS) and mOS [11.7 *versus* 8.1 months; hazard ratio (HR), 0.64; 95% confidence interval (CI), 0.52–0.80; *p* < 0.001].^44^ Recently, the results of the practice changing TOPAZ-1 phase III trial were published that showed for the first time the clinical efficacy and value of immunochemotherapy consisting of the immune checkpoint inhibitor durvalumab with the previous standard chemotherapy gemcitabine + cisplatin in first-line *versus* gemcitabine + cisplatin. The immunochemotherapy led to a significantly better mPFS [7.2 *versus* 5.7 months, HR 0.75 (0.63–0.89)] and mOS [12.8 *versus* 11.5 months, HR 0.80 (0.66–0.97)] and higher OS rates at 24 months with an improvement of 14.5% (24.9 *versus* 10.4% estimated OS at 24 months).^45^

Based on these results, durvalumab was approved by both FDA and EMA for locally advanced or metastatic BTC.^45^ The efficacy of immunotherapy was confirmed by the phase III KEYNOTE-966 trial (NCT04003636) that tested pembrolizumab in conjunction with gemcitabine and cisplatin against gemcitabine and cisplatin + placebo chemotherapy-naïve BTC patients are 1:1 randomized to receive either.^46,47^

The German, open-label, 1:1 randomized, phase II NIFE trial (NCT03044587) compared the platinum-free combination nanoliposomal irinotecan (nal-IRI) + 5-fluorouracil (5-FU)/folinic acid (leucovorin) in arm A *versus* the standard therapy gemcitabine + cisplatin in arm B for treatment-naïve BTC patients. The NIFE trial met its primary endpoint with a PFS-rate of 51% at 4 months in the ITT population (arm A). Overall response rate (ORR) was 24.5% *versus* 11.9%.^48^

The addition of nab-paclitaxel to gemcitabine + cisplatin *versus* gemcitabine + cisplatin and placebo was tested in the phase III randomized trial SWOG S1815 (NCT03768414) in newly diagnosed advanced BTC, however failed to show a statistically significant improvement in mOS for the triple combination.

The data regarding anti-EGFR strategies for BTC management are contradictory. The three randomized phase II trials BINGO, PICCA, and Vecti-BIL and the non-randomized phase II trial T1210 tested the anti-EGFR monoclonal antibodies cetuximab or panitumumab in combination with gemcitabine and a platinum-based chemotherapy in KRAS mutation status-stratified BTC patients; however, these agents failed to prolong mOS.^49–53^ In contrast, two non-randomized phase II trials suggested a potential benefit of anti-EGFR therapies.^54,55^

Regarding the use of the combined use of immunotherapy and anti-VEGF therapy, the randomized phase II trial IMbrave 151 tested atezolizumab in conjunction with bevacizumab and chemotherapy against atezolizumab + placebo and chemotherapy in patients with advanced BTC. However, this trial did not meet its primary endpoint of PFS.^56^

Furthermore, there are ongoing biomarker-guided phase III trials that will be discussed in the respective sections of the review.

Table 3 gives a short overview of trials of first-line treatment for BTC.

**Table 3.** Trials of first-line systemic treatment for BTC.

Trial code Phase Study location Primary trial completion date	Substance	Primary endpoint, Patient number, and Study design	Outcome
NCT03046862; Phase II, South Korea, Study completion: December 2021	Gemcitabine + cisplatin (Biomarker cohort) *versus* Durvalumab + gemcitabine + cisplatin (3C) *versus* Durvalumab + tremelimumab + gemcitabine + cisplatin (4C)	Primary endpoint: ORR, Enrollment: 121; Single group assignment, Open-label	Biomarker cohort: ORR: 50.0%, DCR: 96.7%, mPFS: 13.0 months, mOS: 15.0 months 3C: ORR: 73.4%, DCR: 100%, mPFS: 11.0 months, mOS: 18.1 months 4C: ORR: 73.7%, DCR: 97.8%, mPFS: 11.9 months, mOS: 20.7 months
NCT04238637; Phase II, Germany, Study completion: December 2023	Durvalumab + tremelimumab + specific internal radiotherapy	Primary endpoint: ORR, Enrollment: 50; Parallel assignment, Open-label, Randomized	Not reported
NCT00262769 – ABC-02, phase III, Global, August 2008	Gemcitabine + cisplatin *versus* Gemcitabine	Primary endpoint: mOS, Enrollment: 206 *versus* 204, Parallel assignment, Open-label, Randomized	ORR: 26.1% *versus* 15.5%, DCR: 81.4% *versus* 71.8%, mPFS: 8.0% *versus* 5.0 months, mOS: 11.7% *versus* 8.1 months
NCT04203160 – BilT-04 Phase Ib/II, USA, June 2024	Devimistat + gemcitabine + cisplatin	Primary endpoint: ORR, Estimated enrollment: 78; Sequential assignment, Open-label, Randomized	Not reported
NCT03875235 – TOPAZ-1 Phase III, Global, August 2021	Durvalumab + gemcitabine + cisplatin *versus* Placebo + gemcitabine + cisplatin	Primary endpoint: mOS, Estimated enrollment: 757; Parallel assignment, Quadruple masking, Randomized	ORR: 26.7% *versus* 18.7%, DCR: 85.4% *versus* 82.8%, mPFS: 7.2 *versus* 5.7 months, mOS: 12.9 *versus* 11.3 months, OS rate at 24 months: 23.6% *versus* 11.5%
NCT03473574 – IMMUCHEC, Phase II, Germany, September 2022	5 arms: A: Durvalumab + Tremelimumab (Regimen 1) and Gemcitabine B: Durvalumab + Tremelimumab (Regimen (1) and Gemcitabine and Cisplatin C: Gemcitabine + cisplatin D: Durvalumab + Tremelimumab (Regimen (2) and Gemcitabine E: Durvalumab and Gemcitabine + Cisplatin	Primary endpoint: ORR, Enrollment: A: 22 B: 22 C: 35 D: 30 E: 29; Parallel assignment, Open-label	ORR, mPFS and mOS in 5 arms: A: 4.6%, 2.75 and 7.38 months, B: 18.2%, 5.98 and 12.32 months, C: 28.6%, 8.7 and 16.93 months, D: 26.7%, 8.13 and 22.73 months, E: 20.7%, 5.97 and 12.87 months →Trial missed ORR as primary endpoint
NCT03046862 – MEDITREME, Phase II, South Korea, May 2020	3 groups: -Gemcitabine + cisplatin followed by Gemcitabine + cisplatin and durvalumab + tremelimumab -Gemcitabine + cisplatin + durvalumab -Gemcitabine + cisplatin and durvalumab + tremelimumab	Primary endpoint: ORR, Enrollment: 121; Single group assignment, Open-label	ORR: 50% *versus* 72% *versus* 70%, DCR: 97% *versus* 100% *versus* 98%, mDOR: 9.4 *versus* 11.4 *versus* 8.2 months, mPFS: 12.8 *versus* 11.8 *versus* 12.3 months, mOS: 15.0 *versus* 20.2 *versus* 18.7 months
NCT02591030 – AMEBICA Phase II/III, France, June 2018	FOLFIRINOX *versus* gemcitabine+ cisplatin	Primary endpoint: 6-month PFS rate, Enrollment: 92 *versus* 93, Parallel assignment, Open-label	ORR: 25% *versus* 19.4%, DCR: 66.3% *versus* 69.9%, 6-month PFS rate 44.6% *versus* 47.3%, mPFS: 6.2 *versus* 7.4 months, mOS: 11.7 *versus* 13.8 months, → Trial did not meet the primary study end point
NCT03311789, Phase II, China, October 2018	Nivolumab + gemcitabine + cisplatin	Primary endpoint: PFS rate at 6 months, Enrollment: 32; Single group assignment, Open-label	ORR: 55.6% (37% partial responses), DCR: 92.6%, mPFS: 6.1 months, mOS: 8.5 months
NCT04003636 – KEYNOTE-966, Phase III, Global, August 2023	Pembrolizumab + gemcitabine + cisplatin *versus* Placebo + gemcitabine + cisplatin	Primary endpoint: OS, Estimated enrollment: 1048; Single group assignment, Open-label	ORR. 29% *versus* 29% DCR: 75% *versus* 76% mDOR: 9.7 *versus* 6.9 months mPFS: 6.5 *versus* 5.6 months mOS: 12.7 *versus* 10.9 months
SWOG S1815 (NCT03768414) Phase III, Global, January 2023	Nab-paclitaxel to gemcitabine + cisplatin *versus* gemcitabine + cisplatin and placebo	Primary endpoint OS, Enrollment: 441 Randomized 2:1, Parallel assignment, Open-label	ORR: 34% *versus* 25% mPFS: 8.2 *versus* 6.4 months mOS: 14.0 *versus* 12.7 months
NCT04961788, Phase II, China, December 2021	Toripalimab + gemcitabine + oxaliplatin	Primary endpoint: ORR, Estimated enrollment: 30, Single group assignment Open-label	Not reported
NCT04361331, Phase II, China, December 2021	Toripalimab + lenvatinib	Primary endpoint: ORR, Enrollment: 60, Single group assignment Open-label	ORR: 32.3% DCR: 74.2% Median follow-up: 6.9 months mPFS: not reached mOS: not reached 6-months OS rate: 87.1% DOR: not reached
NCT04361331, Phase II, China, July 2021	Lenvatinib + gemcitabine + oxaliplatin	Primary endpoint: ORR, Enrollment: 60, Single group assignment, Open-label	ORR: 30.0% DCR: 86.7% Median follow-up: 6.8 months mPFS: not reached mOS: not reached 6-months OS rate: 92.6% DOR: not reached
NCT05342194, Phase III, China, May 2027	Toripalimab, lenvatinib, and gemcitabine-based chemotherapy *versus* Toripalimab, oral placebo, and gemcitabine-based chemotherapy *versus* Intravenous placebo, oral placebo, and gemcitabine-based chemotherapy	Primary endpoint: OS, Enrollment: 480 Parallel assignment, Quadruple masking, Randomized	Not reported
NCT01389414 – Vecti-BIL, Phase II, Italy, September 2013	Panitumumab + gemcitabine + oxaliplatin *versus* Gemcitabine + oxaliplatin for KRAS-wildtype BTC	Primary endpoint: PFS, Enrollment: 45 *versus* 44, Parallel assignment, Open-label, Randomized	mPFS: 5.3 *versus* 4.4 months, mOS: 9.9 *versus* 10.2 months, ORR: 26.7% *versus* 18.2%
NCT01320254 – PiCCA, Phase II, Germany, October 2009	Panitumumab + gemcitabine + cisplatin *versus* Gemcitabine + cisplatin	Primary endpoint: 6-month PFS rate, 62 *versus* 28, Parallel assignment, Open-label, 2:1 Randomized	6-month PFS rate: 54% *versus* 73%, ORR: 39% *versus* 45%, DCR: not reported mOS: 20.1 *versus* 12.8 months, PFS: 6.47 *versus* 8.25 months
NCT00552149 – BINGO, Phase II, France and Germany, April 2012	Cetuximab + gemcitabine + oxaliplatin *versus* Gemcitabine + oxaliplatin	Primary endpoint: 4-month PFS rate, 76 *versus* 74, Parallel assignment, Open-label, 1:1 Randomized	4-month PFS rate: 64% *versus* 54%, ORR: 24% *versus* 23%, mDOR: 5.7 *versus* 8.4 months, DCR: 82% *versus* 65%, mOS: 11.0 *versus* 12.4 months, PFS: 6.1 *versus* 5.5 months
NCT01216345 Phase II, Austrian, October 2009	Cetuximab + gemcitabine + oxaliplatin	Primary endpoint: ORR, 30, Single group assignment, Open-label	ORR: 63%, DCR: 80%, mPFS: 8.8 months, mOS: 15.2 months,
EudraCT No. 2006–001694-23 Phase II, Hungary 2006	Cetuximab + gemcitabine + oxaliplatin	Primary endpoint: ORR, 34, Single group assignment, Open-label	ORR: 17.6%, mPFS: mPFS: 34.3 weeks mOS: 34.3 weeks
NCT01267344 – T1210 Phase II Taiwan December 2013	Cetuximab + gemcitabine + oxaliplatin *versus* Gemcitabine + oxaliplatin for KRAS mutation status-stratified	Primary endpoint: ORR, Enrollment: 62 *versus* 60, Parallel assignment, Open-label, Randomized	mPFS: 6.7 *versus* 4.1 months, mOS: 10.6 *versus* 9.8 months, ORR: 27% *versus* 15%
NCT04527679 Phase II China June 2021	Lenvatinib + gemcitabine + cisplatin	Primary endpoint: ORR, Estimated enrollment: 40, Single group assignment Open-label	Not reported
NCT03044587 – NIFE Phase II Germany July 2022	Nal-IRI + 5-FU + folinic acid *versus* Gemcitabine + cisplatin	Primary endpoint: PFS, Enrollment: 49 *versus* 42, Parallel assignment, Open-label, Randomized	PFS rate at 4 months: 51.0% *versus* 59.5% ORR: 24.5% *versus* 11.9% mPFS: 5.98 *versus* 6.87 months -mPFS in iCCA: 3.45 *versus* 7.72 months -mPFS in eCCA: 9.59 *versus* 1.76 months mOS: 15.9 *versus* 13.63 months -mOS in iCCA: 14.19 *versus* 16.36 months -mOS in eCCA: 18.23 *versus* 6.34 months DCR at 2 months: 57.1% *versus* 57.1%
NCT02591030 – AMEBICA Phase II France June 2018	mFOLFIRINOX *versus* Gemcitabine + cisplatin	Primary endpoint: 6-month PFS rate, Enrollment: 92 *versus* 93, Parallel assignment, Open-label, Randomized	6-month PFS rate 44.6% *versus* 47.3% mPFS: 6.2 *versus* 7.4 months mOS: 11.7 *versus* 13.8 months
NCT05290116 Phase 2 China May 2023	HAIC + tislelizumab + apatinib for unresectable iCCA	Primary endpoint: ORR, Estimated enrollment: 17, Single group assignment, Open-label	Not reported
NCT05348811 Phase II China July 2023	HAIC + donafenib + sintilimab for unresectable iCCA	Primary endpoint: ORR, Estimated enrollment: 32, Single group assignment, Open-label	Not reported
NCT05400902 Phase II China May 2023	HAIC + bevacizumab + sintilimab for unresectable iCCA	Primary endpoint: ORR, Estimated enrollment: 17, Single group assignment, Open-label	Not reported
NCT04954781 Phase II China May 2023	TACE + tislelizumab for unresectable iCCA	Primary endpoint: ORR, Estimated enrollment: 25, Single group assignment, Open-label	Not reported
NCT04299581 Phase II China May 2023	Cryoablation + camrelizumab for unresectable iCCA	Primary endpoint: ORR, Estimated enrollment: 25, Single group assignment, Open-label	Not reported
NCT05010668 Phase II China August 2024	Cryoablation + sintilimab + lenvatinib for unresectable iCCA	Primary endpoint: ORR, Estimated enrollment: 25, Single group assignment, Open-label	Not reported
NCT03656536 – FIGHT-302 Phase III Global October 2027	Pemigatinib *versus* Gemcitabine + Cisplatin	Primary endpoint: PFS, Enrollment: 217 *versus* 217, Parallel assignment, Open-label, Randomized	Not reported
NCT04093362 – FOENIX-CCA3 Phase III Global April 2027	Futibatinib *versus* Gemcitabine + cisplatin	Primary endpoint: PFS, Enrollment: 108 *versus* 108, Parallel assignment, Open-label, Randomized	Not reported
NCT03773302 – PROOF Phase III Global January 2026	Infigratinib *versus* Gemcitabine + cisplatin	Primary endpoint: PFS, 2 Enrollment: 00 *versus* 100, Parallel assignment, Open-label, Randomized	Not reported
NCT04430738 – SGNTUC-024, Phase I/II, USA and Japan, May 2024	Tucatinib + trastuzumab and oxaliplatin-based chemotherapy or pembrolizumab-containing combinations for HER2+ gastrointestinal cancers, including CCC	Primary endpoint: safety and efficacy, Enrollment: 120 for the whole cohort, Sequential assignment, Open-label	Not reported

BTC, biliary tract cancer; DCR, disease control rate; eCCA, extrahepatic cholangiocarcinoma; 5-FU, 5-fluorouracil; HAIC, hepatic arterial infusion chemotherapy; iCCA, intrahepatic cholangiocarcinoma; mOS, median overall survival; mPFS, median progression-free; nal-IRI, nanoliposomal irinotecan; ORR, objective response rate; PR, partial response; SD, stable disease; TACE, trans-arterial chemo-embolization.

### SACT in second line without biomarker guidance

Currently – regardless of the subentity – the standard of care in second line is FOLFOX (combination of folinic acid, 5-FU, and oxaliplatin) after failure of gemcitabine + cisplatin established by the ABC-06 trial, the only phase III trial in second line.^57^

However, the application of FOLFOX does not seem always practicable in BTC patients, who did not primarily respond to the platinum-based first-line treatment and who have experienced adverse events, including peripheral polyneuropathy.

Alternative platinum-free treatment options were also tested in second line for BTC.

Two phase II trials tested the efficacy of nal-IRI combined with 5-FU and folinic acid in the interventional arm *versus* 5-FU and folinic acid in the control arm after failure of gemcitabine + cisplatin: The NIFTY trial was conducted in South Korea and the interventional arm achieved an mPFS of 7.1 *versus* 1.4 month and an mOS of 8.6 *versus* 5.5 months.^58^ In contrast, in the German NALIRICC trial, the interventional arm achieved a shorter mPFS of 2.64 *versus* 2.30 months and a shorter mOS of 6.90 *versus* 8.21 months.^59^ The recently presented phase II NAPOLI-2 trial is a single-arm trial, which tested nal-IRI combined with 5-FU and folinic acid at oncologic centers in USA. Similarly to the NIFTY trial, this trial observed a therapeutic efficacy of this therapy regime, achieving a mPFS of 3.9 months and a mOS of 9.5 months.^60^ Thus, the data on the use of nal-IRI are contradictory and more trials are needed in this regard.

The role of immunotherapy in second line was investigated in three phase II trials: CA209-538, LEAP-005, and REGOMUNE. The Australian trial CA209-538 examined the chemotherapy-free combination regimen of ipilimumab and nivolumab and yielded an ORR of 23% (all partial responses), however with a relatively modest mPFS of 2.9 months and mOS of 5.7 months.^61^

The combination of lenvatinib + pembrolizumab in LEAP-005 yielded an ORR of 10% (all partial responses) and a mPFS of 6.1 months and a mOS of 8.6 months.^62^ REGOMUNE investigated regorafenib + avelumab that led to promising results, including an ORR of 14% (all partial responses), a mPFS of 2.5 and a mOS of 11.9 months.^63^

Different tyrosine kinase inhibitors (TKIs) were studied in different phase II trials, including regorafenib (MCC-17651 and REACHIN), apatinib (JS-1392), sunitinib (SUN-CK), and varlitinib (TreeTopp).^64–68^ Regorafenib, apatinib, and sunitinib demonstrated clinical efficacy.^64–67^ The TreeTopp trial failed to significantly improve the mPFS.^68^ Table 4 gives a short overview of trials for second-line BTC treatment.

**Table 4.** Trials after at least one prior line of systemic therapy for BTC.

Trial code Phase Study location Primary trial completion date	Substance	Primary endpoint, Patient number and Study design	Outcome
NCT02456714 – 4CC, Phase II, Netherlands	FOLFIRINOX in irresectable cholangiocarcinoma	Primary endpoint: ORR, Estimated enrollment: 30, Single group assignment, Open-label	ORR: 10%, DCR: 67%, mPFS: 6.2 months, mOS: 10.7 months
NCT04059562 – TRITICC, Phase II, German, October 2023	Trifluridine/tipiracil + Irinotecan	Primary endpoint: PFS, Estimated enrollment: 28, Single group assignment, Open-label	Not reported
NCT01926236 – ABC-06, Phase III, Global, January 2018	mFOLFOX *versus* Active symptom control	Primary endpoint: OS, Enrollment: 81 *versus* 81, Parallel assignment, Open-label	ORR: 5%, DCR: 33%, mPFS: 4.0 months, mOS: 6.2 *versus* 5.3 months
NCT03524508 – NIFTY, Phase IIb, South Korea, September 2020	Nal-IRI + 5-FU/LV *versus* 5-FU/LV	Primary endpoint: PFS, Enrollment: 88 *versus* 86, Parallel assignment, Open-label, 1:1 randomized	ORR: 14.8% *versus* 5.8% (all partial responses) DCR: 64.8 *versus* 34.9% mPFS: 7.1 *versus* 1.4 month mOS: 8.6 *versus* 5.5 months
NCT03043547 – NALIRICC, Phase II, Germany, December 2021	Nal-IRI + 5-FU/LV *versus* *5-FU/LV*	Primary endpoint: PFS, Enrollment: 49 *versus* 51, Parallel assignment, Open-label, 1:1 randomized	ORR: 5% DCR: 33% mPFS: 4.0 months mOS: 6.2 *versus* 5.3 months
NCT04005339 – NAPOLI-2, Phase II, USA, November 2023	Nal-IRI + 5-FU/LV	Primary endpoint: PFS, Enrollment: 25 Single group assignment, Open-label	ORR: 16% DCR: 52% mPFS: 3.9 months PFS rate at 4 months: 46% mOS: 9.5 months
NCT03797326 – LEAP-005, Phase II, Global, December 2023	Pembrolizumab + lenvatinib in different tumors, including BTC	Primary endpoint: ORR, Enrollment: 31, Parallel assignment, Open-label, Non-randomized	ORR: 5% DCR: 33% mPFS: 4.0 months mOS: 6.2 *versus* 5.3 months
NCT02923934 – CA209-538, Phase II, Australia, April 2020,	Ipilimumab + Nivolumab in different tumors, including BTC	Primary endpoint: DCR, Enrollment: 39, Single group assignment, Open-label	ORR: 23% (all partial responses), DCR: 44%, mPFS: 2.9 months, mOS: 5.7 months
NCT02115542 – MCC-17651, Phase II, USA, December 2018	Regorafenib	Primary endpoint: 39, Enrollment: 43, Single group assignment, Open-label	ORR: 11% (all partial responses) DCR: 56% mPFS: 15.6 weeks (approximately 3.9 months) mOS: 31.8 weeks (approximately 7.95 months)
NCT02162914 – REACHIN, Phase II, Belgium, December 2018	Regorafenib *versus* Placebo	Primary endpoint: 66, Enrollment: 33 *versus* 33, Parallel assignment, Triple masking, 1:1 randomized	ORR: not reported DCR: not reported, mPFS: 3.0 *versus* 1.5 months mOS: 5.3 *versus* 5.1 months
NCT03475953 – REGOMUNE, Phase I/II, France, December 2020	Regorafenib + avelumab in solid tumors, including BTC	Primary endpoint: ORR, Enrollment: 29, Sequential assignment, Open-label	ORR: 14% (all partial responses) DCR: 52% mDOR: 10.4 months mPFS: 2.5 months, mOS: 11.9 months
NCT03704480 – IMMUNOBIL – D18-1 PRODIGE 57, Phase II, France, October 2022	Durvalumab + tremelimumab without (Arm A) or with weekly paclitaxel (Arm B)	Primary endpoint: OS, Enrollment: 106 in Arm A Single group assignment, Open-label, 1:1 randomized	Arm B closed prematurely due to toxicity, Arm A: ORR: 9.7% (7.8% had partial responses) DCR: 40.8% mPFS: 2.5 months mOS: 8.0 months mOS rate at 6 months: 59.2%,
NCT03251443 – JS-1392 Phase II China March 2019	Apatinib	Primary endpoint: PFS, Enrollment: 26, Single group assignment, Open-label	ORR: 11.5% (all partial responses) DCR: 50.0% mPFS: 2.0 mOS: 9.0
NCT03093870 – TreeTopp Phase II Global November 2019	Varlitinib + capecitabine *versus* Placebo + capecitabine	Primary endpoint: ORR and PFS, Enrollment: 64 *versus* 63, Parallel assignment, Double-blind, Randomized	ORR: 9.4 *versus* 4.8%, mPFS: 2.83 *versus* 2.79 months, mOS: 7.8 *versus* 7.5 months
NCT01718327 – SUN-CK Phase II France November 2016	Sunitinib monotherapy	Primary endpoint: OS, Enrollment: 34, Single group assignment, Open-label	ORR: 15% (all partial responses) SD: 71% DCR: 85% mPFS: 5.2 months mOS: 9.6 months
NCT02989857 – ClarIDHy Phase III Global January 2019	Ivosidenib in patients with IDH1 mutations *versus* Placebo	Primary endpoint: PFS, Enrollment: 124 *versus* 61, Parallel assignment, Double-blind, 2:1 Randomized	ORR: 2% (all partial responses) *versus* 0% DCR: 51 *versus* 28% mPFS: 2.7 *versus* 1.4 month mOS: 10.3 *versus* 7.5 months, mOS (adjusted for crossover): 10.3 *versus* 5.1 months
NCT03212274 Phase II USA July 2023	Olaparib in IDH1 or IDH2 mutant tumors, including CCC	Primary endpoint: ORR, Estimated enrollment: 145 for total cohort Single group assignment, Open-label	Not reported
NCT03878095 Phase II USA March 2023	Olaparib + Ceralasertib in IDH1 or IDH2 mutant tumors, including CCC	Primary endpoint: ORR, Estimated enrollment: 50 for total cohort, Single group assignment, Open-label	Not reported
NCT02428855 Phase II USA February 2018	Dasatinib in patients with IDH1 mutations	Primary endpoint: ORR, Enrollment: 8, Single group assignment, Open-label	ORR: 0% DCR: not reported mPFS: 8.7 weeks (approximately 2.2 months) mOS: 37.9 weeks (approximately 9.5 months)
NCT02924376 – FIGHT-202 Phase II Global February 2022	Pemigatinib (FGFR1-3 inhibitor) in patients with -FGFR2 fusions or rearrangements *versus* -other FGF/FGFR alterations *versus* -negative for FGF/FGFR alteration	Primary endpoint: ORR, Enrollment: 107 *versus* 20 *versus* 18, Parallel assignment, Open-label, Non-randomized	-FGFR2 fusions or rearrangements: mPFS: 6.9 months, mOS: 21.1 months DCR: 82.2%, ORR: 35.5% mDOR: 7.5 months -Other FGF/FGFR alterations: mPFS: 2.1 months, mOS: 6.7 months DCR: 40.0%, ORR: 0% -Negative for FGF/FGFR alteration: mPFS: 1.7 months, mOS: 4 months DCR: 22.2%, ORR: 0%
NCT04256980 Phase II China June 2022	Pemigatinib (FGFR1-3 inhibitor) in patients with FGFR2 fusions or rearrangements	Primary endpoint: ORR Enrollment: 30, Single group assignment, Open-label	ORR: 50% DCR: 100% mDOR: not reached mPFS: 6.3 months mOS: not reported
NCT03230318 – FIDES-01 Phase I/II Global September 2022	Derazantinib (FGFR1-3 inhibitor) in patients with -FGFR2 fusions or rearrangements -FGFR2 mutations or amplifications	Primary endpoint: ORR, Enrollment: 28, Single group assignment, Open-label	- FGFR2 fusions or rearrangements ORR: 20.7% DCR: 82.8% mPFS: 5.7 months mOS: not reached -FGFR2 mutations or amplifications ORR: 8.7% DCR: 73.9% mPFS: 7.3 months mOS: not reported
NCT02150967 Phase II Global July 2022	Infigratinib (FGFR1-4 inhibitor) in patients with FGFR2 alteration	Primary endpoint: ORR, Enrollment: 108–77% had FGFR2 fusion	ORR: 23.1% -ORR in second line: 34% -ORR in ⩾ line: 13.8% DCR: not reported mDOR: 5.0 months, mPFS: 7.3 months,
NCT02052778- FOENIX-CCA2 Phase II Global May 2021	Futibatinib (irreversible FGFR1-4 inhibitor) in patients with FGFR2 fusions or rearrangements	Primary endpoint: ORR, Enrollment: 67, Sequential assignment, Open-label, Non-randomized	ORR: 41.7% DCR: 82.5% mDOR: 9.1 months mPFS: 8.9 months 12-months PFS rate: 35.4% mOS: 20.0 months 12-months OS rate: 73.1%
NCT02699606 Phase IIb Asian population October 2021	Erdafitinib (FGFR1-4 inhibitor) in patients with FGFR2 alteration	Primary endpoint: ORR, Enrollment: 14–57% had FGFR2 fusion, Single group assignment, Open-label	ORR: 50.0% DCR: 83.3% mDOR: 6.83 months mPFS: 5.59 months
NCT04083976 – RAGNAR Phase II Global December 2027	Erdafitinib (FGFR1-4 inhibitor) in patients (both adults and pediatric) with FGFR alterations	Primary endpoint: ORR, Estimated enrollment: 31, Single group assignment, Open-label	Interim Analysis ORR: 41.9% mDOR: not reported DCR: not reported mPFS: not reported mOS: not reported
NCT04526106 – REFOCUS Phase I/II Global April 2024	RLY-4008 (Selective FGFR2 Inhibitor) in patients with FGFR2 fusion or rearrangement	Primary endpoint: ORR, Enrollment: 38, Parallel assignment, Open-label	ORR (confirmed): 57.9% DCR: 94.7%, mDOR: not reported mPFS: not reported mOS: not reported
NCT02034110 – ROAR Phase II Global December 2021	Dabrafenib–Trametinib in BRAF V600E mutant tumors	Primary endpoint: ORR, Enrollment: 43, Single group assignment, Open-label	ORR: 56% DCR: 82%, mDOR: 14.4 months mPFS: 6.7 months mOS: 14.5 months
NCT01953926 – SUMMIT, Phase II, Global, October 2022	Neratinib in HER2 mutant BTC	Primary endpoint: ORR, Enrollment: 25, Parallel assignment, Open-label	ORR: 16% (all partial responses) DCR: 28% mPFS: 2.8 months mOS: 5.4 months
NCT02999672 – KAMELEON, Phase II, Global, April 2018	Trastuzumab Emtansine in HER2 overexpressing solid tumors, including CCC	Primary endpoint: ORR, Enrollment: 20, Parallel assignment, Open-label	Not reported
NCT02091141 – MyPathway, Phase IIa, USA, January 2023	Trastuzumab + pertuzumab in HER2-positive BTC	Primary endpoint: ORR, Enrollment: 39, Parallel assignment, Open-label	ORR: 23% (all partial responses) DCR: 51%, mDOR: 10.8 months mPFS: 4.0 months mOS: 10.9
NCT04722133 – KCSG-HB19-14 Phase II, South Korea, September 2022	Trastuzumab + mFOLFOX	Primary endpoint: ORR, Enrollment: 34, Parallel assignment, Open-label	ORR: 29.4% DCR: 79.4% mPFS: 5.1 months mOS: not reached
JMA-IIA00423 – HERB, Phase II, Japan, June 2023	Trastuzumab deruxtecan in HER2-positive BTC	Primary endpoint: ORR, Enrollment: 30, Single group assignment, Open-label	ORR: 36.4% DCR: 81.8, mPFS: 4.4 months mOS: 7.1 months
NCT04482309, Phase II, Global, June 2023	Trastuzumab deruxtecan in HER2-positive tumors, including BTC – DESTINY-PanTumor02	Primary endpoint: ORR, Enrollment: 16 BTC patients Parallel assignment, Open-label	ORR: 56.3% DCR: not reported mPFS: not reported mOS: not reported
NCT00107536, Phase II USA, May 2009	Lapatinib in molecular unselected BTC and HCC patients	Primary endpoint: ORR, Enrollment: 17 BTC patients, Single group assignment, Open-label	ORR: 0% DCR: 26% mPFS: 1.8 months mOS: 5.2 months
No trial code (DOI: 10.1159/336,488), Phase II, USA, Early termination due to possible futility in June 2006	Lapatinib in BTC patients without EGFR or HER2 mutations and without HER2 overexpression	Primary endpoint: ORR, Enrollment: 9 patients, Single group assignment, Open-label	ORR: 0% DCR: 50% Mean PFS: 2.6 months, Mean OS: 5.1 months
NCT04329429, Phase II, China, August 2023	Disitamab vedotin (ADC) in HER2-positive BTC patients	Primary endpoint: ORR, Estimated enrollment: 57, Single group assignment, Open-label	Not reported
NCT05540483 – RIGHT, Phase II, China, August 2024	Disitamab vedotin (ADC) + Zimberelimab (PD-1 inhibitor) in HER2-positive BTC patients	Primary endpoint: ORR, Estimated enrollment: 31, Single group assignment, Open-label	Not reported
NCT04466891-HERIZON-BTC-01, Phase IIb, Global December 2022	Zanidatamab (bispecific antibody) in HER2-positive BTC patients	Primary endpoint: ORR, Estimated enrollment: 100, Single group assignment, Open-label	ORR: 41.3% mDOR: 12.9 months mPFS: 5.5 months mOS: immature
NCT04837508, Phase II, China, December 2022	MRG002 (ADC) in HER2-positive BTC patients	Primary endpoint: ORR, Estimated enrollment: 86, Single group assignment, Open-label	Not reported
NCT03185988, Phase II, China, July 2017	Trastuzumab + chemotherapy in HER2-positive tumors, including BTC	Primary endpoint: ORR, Estimated enrollment: 100, Parallel assignment, Open-label	Not reported
NCT04579380 – SGNTUC-019 Phase II, Global, May 2023	Tucatinib + trastuzumab in HER2-positive tumors, including BTC	Primary endpoint: ORR, Enrollment: 30 BTC patients, Parallel assignment, Open-label	ORR: 46.7% mDOR: 6.0 months DCR: 76.7% mPFS: 5.5 months mOS: not reported 12-months OS rate: 53.8%
NCT05222971 – OPTIMUM, Phase II, South Korea, October 2023	Olaparib with or without durvalumab for DDR gene mutated BTC	Primary endpoint: PFS rate at 6 months, Estimated enrollment: 62, Parallel assignment, Open-label, 1:1 randomized	Not reported
NCT04042831, Phase II, USA, September 2022	Olaparib for DDR gene mutated BTC	Primary endpoint: ORR, Estimated enrollment: 100, Single group assignment, Open-label	Not reported
NCT02628067 – KEYNOTE-158, Phase II, Global, June 2026	Pembrolizumab MSI-H TMB-H	Primary endpoint: ORR, Estimated enrollment: 104 BTC patients, Parallel assignment, Open-label	ORR: 5.8%, mDOR: not reached mPFS: 2.0 months mOS: 7.4 months
CodeBreak100, Phase I/II, Global, May 2027	Sotorasib in KRAS G12C mutated solid tumors	Primary endpoint: ORR, Enrollment: 129 patients, one patient had BTC, Sequential assignment, Open-label	Data not usable – only one patient had BTC
NCT03785249 – KRYSTAL-1, Phase I/II, USA, December 2023	Adagrasib in KRAS G12C mutated solid tumors	Primary endpoint: ORR, Enrollment: 12 BTC patients, Sequential assignment, Open-label	ORR: 41.7% DCR: 91.7% mPFS: 8.6 months mOS: 15.1 months

ADC, antibody–drug conjugate; BTC, biliary tract cancer; DCR, disease control rate; DDR, DNA damage response; eCCA, extrahepatic cholangiocarcinoma;; 5-FU, 5-fluorouracil; HAIC, hepatic arterial infusion chemotherapy; iCCA, intrahepatic cholangiocarcinoma; mOS, median overall survival; mPFS, median progression-free survival; nal-IRI, nanoliposomal irinotecan; ORR, objective response rate; PD1, Programed cell death protein 1; PR, partial response; SD, stable disease

Table 4 gives a short overview of ongoing trials.

### Targeted treatments in palliative setting

In recent years, efforts have been made to progressively individualize therapy options in specific cancers. Emerging techniques, such as profiling tumor molecular alterations and mutations, identifying molecular targets amenable to specific treatments, and developing drug treatments specific to an individual patient, have created the potential for novel and effective therapies.^69,70^ The most common mutations in BTC are TP53, KRAS, CDKN2A/B, and SMAD4.^71^ None of these mutations are currently targetable, except for KRAS G12C that can be targeted off-label with Sotorasib.^72^ In this work, the genetic aberrations are ranked and rated according to the ESMO ESCAT (ESMO Scale for Clinical Actionability of molecular Targets) to objectify their value as clinical targets based on available strength of evidence.^73,74^ In a study of 327 BTC patients conducted by Verdaguer *et al.*, 56.3% had actionable molecular aberrations according to ESCAT. Patients who received molecular-based targeted therapy based on their molecular profile had an mOS of 22.6 months, compared to 14.3 months in those without actionable ESCAT alterations.^74^ Thus, targeted therapies based on the molecular information are crucial for improving the clinical outcome.

Table 5 classifies the various targets based on clinical evidence of utility according to the ESMO ESCAT framework.

**Table 5.** Classification of the targets in BTC according to the European Society of Medical Oncology Scale for Clinical Actionability of Molecular Targets (ESMO ESCAT framework) adapted from Biliary tract cancer: ESMO Clinical Practice Guideline for diagnosis, treatment, and follow-up.^75^

Gene/Finding	Type of aberration	ESMO ESCAT	Targeted therapy and approval
IDH1	Mutation	IA	Ivosidenib (approved by FDA)
FGFR2	Fusions or rearrangements	IB	Pemigatinib (FDA and EMA approved), Futibatinib (FDA and EMA approved), Infigratinib (FDA approved), Derazantinib, RLY-4008
BRAF V600E	Mutation	IB	Dabrafenib + trametinib (FDA approved)
MSI-H	Genomic instability	IC	Pembrolizumab (tissue-agnostic approval by FDA and EMA)
TMB-H	Genomic instability	IC	Pembrolizumab (tissue-agnostic approval by FDA and EMA)
NTRK	Fusions	IC	Larotrectinib (tissue-agnostic approval by FDA and EMA), Entrectinib tissue-agnostic approval by FDA and EMA)
RET	Fusion	IC	Selpercatinib (tissue-agnostic FDA approval), Pralsetinib
HER2	Amplification	IC	Trastuzumab + mFOLFOX, Trastuzumab + Pertuzumab, Trastuzumab + Tucatinib, Trastuzumab deruxtecan, Zanidatamab
KRAS G12C	Mutation	IC	Adagrasib Sotorasib
BRCA1/2	Mutations	IIIA	Olaparib
PIK3CA	Mutation	IIIA	Alpelisib
MET	Amplification	IIIA	Capmatinib Tepotinib

BTC, biliary tract cancer.

#### IDH1

IDH1 (isocitrate dehydrogenase 1) mutations are found in approximately 10–15%, predominantly in the iCCA, particularly in the variants small duct-iCCA and cholangiolocarcinoma-iCCA.^76,77^ IDH1 mutations can be targeted by Ivosidenib, which is an oral IDH1 inhibitor that has been evaluated in the global, randomized, double-blind, placebo-controlled phase III clinical ClarIDHy trial enrolling patients with pretreated IDH1 mutant cholangiocarcinoma. The primary endpoint, namely mPFS, was met with a significant improvement of 1.3 month (2.7 *versus* 1.4 month; HR 0.37). An mOS showed a trend toward improvement with Ivosidenib, but it was not statistically significant (10.3 *versus* 7.5 months, HR 0.79). However, when adjusted for crossover, OS was significantly better with Ivosidenib (mOS 5.1 months with placebo *versus* not reached with Ivosidenib.^78^ Ivosidenib received an ESMO-MCBS score of 3 out of 5 points.^79^

Ivosidenib, an oral IDH1 inhibitor, has been studied in a phase III clinical trial (ClarIDHy) for patients with pretreated IDH1 mutant cholangiocarcinoma. The trial demonstrated a significant improvement in mPFS with Ivosidenib compared to placebo (2.7 months *versus* 1.4 months, HR 0.37).

#### FGFR

The FGF pathway is composed of 22 FGFs and 4 different transmembrane receptors with intracellular tyrosine kinase domains, FGFR 1–4.^80^ In this context, gene fusions that involve the FGFR2 are particularly clinically relevant, as they occur in approximately 10–15% of patients with iCCA and can be specifically targeted. Until now, more than 150 fusions partners have been described for FGFR2 fusions.^81^ In recent years, different FGFR-inhibitors have been developed, including pemigatinib, futibatinib, infigratinib, derazantinib, and erdafitinib, and RLY-4008 that have demonstrated promising outcome in several phase II trials enrolling pretreated BTC patients with FGFR alterations.^82–87^ In all these trials, the mPFS was well above 5 months with an ORR of more than 20%.^88,89^ Pemigatinib and futibatinib are the only FGFR-inhibitor that have been granted approval from both FDA and EMA and received an ESMO-MCBS score of 3 from 5 points.^90,91^ All aforementioned inhibitors – except for futibatinib – are reversible inhibitors.^92^

Currently, three large phase III trials are ongoing that investigate pemigatinib, futibatinib, and infigratinib against chemotherapy, respectively, in first line in BTC patients with FGFR2 gene fusions/translocations – with PFS being the primary endpoint.^93–95^

Table 1 gives a short overview of trials of first-line treatment for BTC.

#### BRAF V600E

BRAF is a kinase and part of the RAS–RAF–MEK–ERK pathway, which is a critical signaling cascade in oncogenesis. The most common and the clinically most relevant BRAF mutation is the BRAF V600E point mutation that is detected in 5% in iCCA cases.^96^

In the phase II, non-randomized ROAR basket trial, Subbiah *et al.* investigated the BRAF inhibitor dabrafenib and the MEK inhibitor trametinib in 43 pretreated BTC patients, with the majority (91%) having iCCA and harboring the BRAF V600E mutation. The targeted therapy combination resulted in an ORR of 56%, with an mPFS of 6.7 months, and an mOS of 14.5 months.^97,98^

Recently, dabrafenib + trametinib was approved by the FDA as a tissue-agnostic therapy option for BRAF V600E mutant solid tumors, including BTC.^99^

#### HER2

HER2 (also known as ERBB2) amplification is detected in approximately 5% of all BTC cases, particularly in gallbladder carcinoma and in eCCA, where it is found between 15 and 20%.^100,101^ It is associated with worse OS.^102^ Several clinical phase II trials explored anti-HER2-targeted therapies in pretreated HER2-positive BTC patients that have shown clinical efficacy, including trastuzumab deruxtecan, trastuzumab + mFOLFOX, trastuzumab + pertuzumab, trastuzumab + tucatinib, and zanidatamab. These trials yielded an mPFS between 4.0 and 5.5 months and an ORR of over 20%.^103–107^

In contrast, based on two phase II trials, lapatinib does seem to be very active in BTC.^108,109^

The phase II basket trial SUMMIT is the only trial that investigated the irreversible pan-HER oral TKI neratinib in BTC patients with HER2 mutations and achieved an ORR of 16% and an mPFS of 2.8 months and an mOS of 5.4 months.^110^

#### DDR, MSI, TMB

Aberrations in genes of the DNA damage response (DDR) are found in over 20% BTC patients, with a higher occurrence frequency in eCCA.^111^ The presence of an aberrated DDR is a predictive marker for platinum-based therapies.^112^ These genes include, but are not limited to: BRCA1/2, PALB2, ATM, ATR, RAD51, PTEN, FANCA, FANCAB, MRE11, and ARID1A.^113^ In a retrospective study, Spizzo *et al.* investigated 1292 BTC samples and identified BRCA mutations in 3.6% of all cases and noticed that these mutations were associated with a higher rate in subjects with MSI-H (microsatellite instability-high) (19.5 *versus* 1.7%, *p* < 0.0001) and tumors with higher tumor mutational burden (TMB), regardless of the MSI status (*p* < 0.05).^114^ Due to the concept of synthetic lethality, BRCA deficient tumors are sensitive to therapies with PARP inhibitors.^115^ Currently, two phase II trials explore the clinical efficacy of the PARP inhibitor olaparib in DDR aberrated BTC (see Table 2).

MSI-H is a predictive marker for immunotherapy and is found in below than 5% of BTC patients.^116^ The multicohort phase II trial KEYNOTE-158 included 22 pretreated BTC patients with MSI-H who were treated with pembrolizumab.^117^ An ORR of 40.9% was achieved. The mPFS and mOS were 4.2 and 24.3 months, respectively. The mDOR was not reached.^118^ Based on these results, pembrolizumab received the first tumor tissue-agnostic approval from the FDA for the treatment of MSI-H positive solid tumors in 2017.^119^ Recently, EMA granted approval to MSI-H positive BTC, who have disease progression on or following at least one prior therapy.

Tumors with a high TMB (TMB-H) tend to genomic instability resulting in an increased likelihood of tumor-specific neoantigens recognizable by the immune system. TMB is commonly defined as the overall number of somatic nonsynonymous mutations per megabase (Mut/Mb). TMB-H is defined as 10 mutations/megabase and has been shown to be more susceptible to immunotherapy than tumors with a low TMB. The frequency of TMB-H is reported at approximately 4% of BTC patients.^120^

In 2020, FDA approved pembrolizumab for patients with TMB-H solid tumors based on the KEYNOTE-158 trial.^121^

#### Other markers

Gene fusions involving one of the neurotrophic tropomyosin receptor kinases (NTRK1, NTRK2, and NTRK3) can act as an oncogenic driver. These gene fusions are a rare finding in BTC, typically below 1%.^122,123^

Despite the low incidence of these fusions, they are clinically highly relevant, as they can be targeted by specific TKIs, namely entrectinib and larotrectinib, that both have received approval from FDA and EMA based on different phase I and II trials.^124–127^

Other rare findings occurring in BTC are RET gene fusions that have been investigated and targeted in the two phase I/II basket trials, ARROW and LIBRETTO-001.^128,129^ In the former trial, pralsetinib was tested in three BTC patients with RET fusions and achieved a clinical response in two patients.^128^ In the latter trial, selpercatinib was evaluated in 41 patients, including 2 BTC patients, and achieved an ORR of 44% with a mDOR of 24.5 months.^129^ Based on this trial, FDA granted selpercatinib an approval for locally advanced or metastatic RET fusion–positive solid tumors.^130^

For other markers such as PIK3CA mutations and MET amplifications the evidence for targeted therapies in BTC is scarce.

KRAS G12C, albeit a rare finding (1%), is amenable to targeted therapy.^131^

**Figure 1.**
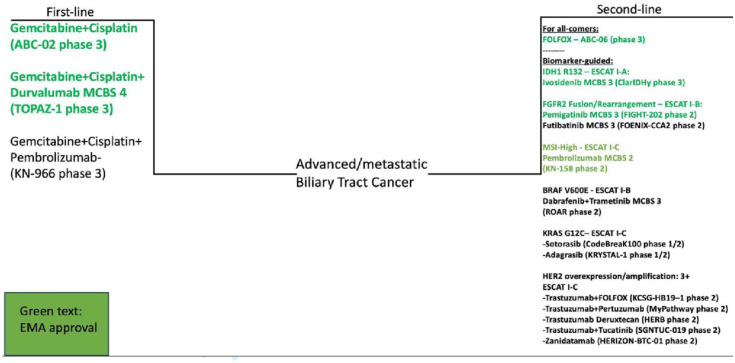
Palliative treatment options for advanced/metastatic Biliary Tract Cancer.

See Table 4 for further details. See Figure 1 for an overview of the current treatments for BTC.

### Locoregional treatments in palliative setting

As unresectable iCCA often manifests in the liver, loco-regional treatments have been utilized in such cases. These treatment techniques encompass a range of approaches, from ablation techniques, to external beam radiotherapy (EBRT) to and intra-arterial therapies (IATs). Among the IAT approaches are trans-arterial chemo-embolization (TACE), SIRT or radioembolization), and hepatic arterial infusion of chemotherapy (HAI), each with distinct mechanisms of action.^132–134^

#### Ablation techniques

Ablation techniques involve minimally invasive procedures aimed at inducing coagulative necrosis within the tumor. Commonly utilized ablation techniques include radiofrequency ablation (RFA), microwave ablation (MWA), and irreversible electroporation (IRE). Ablation procedures have predominantly been studied in patients with tumors that cannot be surgically removed due to cirrhosis or those experiencing tumor recurrence following prior resection.

As reported in a meta-analysis by Edeline *et al.*, ablation was associated with a pooled complete response rate of over 90%, and with a pooled weighted mean OS of over 30 months.^135^ Based on this meta-analysis, the ESMO recommends considering ablation for patients with intrahepatic cholangiocarcinoma (iCCA) of ⩽3 cm in size who have contraindications to surgery.^75^

#### External beam radiotherapy

EBRT involves the delivery of high-energy radiation beams from an external source to the tumor site. Data regarding its use are limited and heterogeneous and should be investigated further in prospective clinical trials.^75^

#### Intra-arterial therapies

IAT approaches involve the targeted delivery of therapeutic agents directly into the hepatic arterial supply of the tumor. The key IAT modalities TACE, SIRT, and HAI have been mostly studied retrospectively. Two meta-analyses supported the use of TACE in iCCA.^136,137^ HAI and SIRT combined with systemic chemotherapy were explored in single-arm phase II trials in iCCA and yielded promising results.^138,139^ Thus, IAT might be considered combined with systemic antitumoral therapy in iCCA limited to the liver in selected cases.^75^

### Role of cirrhosis in advanced intrahepatic cholangiocarcinoma

Based on the European Network for the Study of Cholangiocarcinoma (ENSCCA) registry over 10% of patients with iCCA have cirrhosis.^140^ Cirrhosis has been reported as a risk factor for the development of cholangiocarcinoma.^141^ In advanced iCCA, it is significantly associated with more grade 3/4 chemotherapy-induced toxicities and shorter OS.^142,143^ Thus, early diagnosis, grading and consideration of cirrhosis in therapeutic management, particularly the dose of chemotherapy, is recommended.^142^

## Discussion

Neoadjuvant treatment can convert unresectable BTC to resectable, enabling curative resection attempts. Although neoadjuvant therapy may be suitable for locally advanced disease, its routine use in upfront resectable CCA is not well established, and its impact on survival outcomes needs further investigation. Several issues, such as the timing of surgical resection after neoadjuvant therapy, duration of treatment, safety during preoperative biliary drainage and embolization, and risks of major hepatic resection, require thorough assessment in prospective studies. Large-scale clinical trials are essential to establish a standardized neoadjuvant therapy regimen for both resectable and unresectable CCA. Thus, the authors suggest that neoadjuvant treatment should not be proposed outside of clinical trials and only with histological proof.

Despite surgical resection with curative intention, the risk of recurrence remains high for BTC.

A combined perioperative therapeutic approach is crucial in reducing the relapse rate.

By optimizing treatment sequences and synergizing modalities, a multimodal treatment strategy – as for example pursued in the phase II SIROCHO trial (NCT05265208) – may aid in reducing the chances of recurrence.

After more than a decade of stagnancy in novel treatment options for advanced and metastatic BTC, the molecular profiling has led to treatment stratification, which is has improved the prognosis of the patients, thereby sparing unnecessary toxicities from chemotherapy. Thus, an increasing number of molecular targets have been described and new targeted agents, such as ivosidenib and pemigatinib and futibatinib, have been developed and successfully tested in prospective clinical phase II and III trials and brought new drugs into standard treatment algorithm. Ongoing trials are currently testing these and other targeted agents in the first-line setting of advanced and metastatic BTC.

Furthermore, immune checkpoint inhibitors have been investigated in a variety of entities, including BTC. In 2010, the positive phase III ABC-02 trial set a new standard with the systemic chemotherapy combination with gemcitabine + cisplatin, which was for a long time the mainstay of the BTC therapy. However, the phase III TOPAZ-1 trial established the immune checkpoint inhibitor durvalumab combined with chemotherapy as a first-line treatment and underlined the potential for immunotherapy in BTC.

Thus, both immunotherapy and targeted therapies are shaping and enriching the therapeutic armamentarium of BTC management.

However, there is still room for improvement. For the application of immunotherapy, the identification of new predictive biomarkers – apart from MSI-H and TMB-H – would be crucial to better select and stratify patients that may have a long-term benefit from this therapy type. Another clinical priority is to identify primary and secondary therapeutic resistance mechanisms. Even the use of targeted therapies, such as ivosidenib and pemigatinib, produces an mPFS under 8 months. Thus, to increase the clinical efficacy of therapies, these resistance mechanisms have to be overcome by introducing new potent therapeutics, effective therapy combination, and rational therapy sequence strategies.

Furthermore, the molecular heterogeneity of BTC subentities should be considered in future trials. Multicohort trials covering the different subentities would be one way to increase the accuracy and quality of drug investigation in BTC.

Currently, numerous clinical trials are ongoing, and many more trials are designed that will help to clarify these open questions.

## Declarations

**Ethics approval and consent to participate:** Not applicable.

**Consent for publication:** All authors gave their consent for publication.

**Availability of data and materials:** Not applicable.
